# Effects of microsurgery on short-term serum neurotransmitter levels and long-term cerebral neurological function in elderly patients with spontaneous intracerebral hemorrhage

**DOI:** 10.3389/fneur.2025.1653647

**Published:** 2025-09-03

**Authors:** Yang Zhang, Rujun Pan, Zhengjian Liao, Shaoya Yin

**Affiliations:** ^1^Clinical College of Neurology, Neurosurgery and Neurorehabilitation, Tianjin Medical University, Tianjin, China; ^2^Department of Neurosurgery, Fuzhou University Affiliated Provincial Hospital (Fujian Provincial Hospital), Fuzhou, China; ^3^Department of Neurosurgery, Tianjin Huanhu Hospital, Tianjin, China

**Keywords:** microsurgery, spontaneous intracerebral hemorrhage, neurotransmitters, cerebral neurological function, long-term functional outcomes

## Abstract

**Objective:**

This study explores the impact of microsurgery on postoperative serum neurotransmitter levels and long-term neurological outcomes in elderly spontaneous intracerebral hemorrhage (SICH) patients.

**Methods:**

A single-center, prospective, and single-arm cohort study was conducted, enrolling 106 elderly SICH patients aged ≥60 years who underwent microsurgical hematoma evacuation within 24 h of onset. Serum levels of glutamate (Glu), gamma-aminobutyric acid (GABA), norepinephrine (NE), and serotonin (5-hydroxytryptamine, 5-HT) were measured before surgery and on postoperative day 7. Neurological outcomes were assessed at 180 days using the modified Rankin Scale (mRS) and Glasgow Outcome Scale (GOS). Subgroup analyses were performed based on hematoma volume (≥30 mL vs. <30 mL), hemorrhage location (basal ganglia, thalamus, or lobar), and preoperative Glasgow Coma Scale (GCS) scores (3–8, 9–12, or 13–15). Pearson correlation analysis evaluated the relationship between neurotransmitter levels and outcomes.

**Results:**

On postoperative day 7, Glu and NE levels decreased, while GABA and 5-HT levels increased. Larger hematoma volume, lobar location, and moderate GCS (9–12) were associated with higher Glu/NE, lower GABA/5-HT, and worse mRS/GOS scores. Glu and NE levels positively correlated with mRS and negatively with GOS (*p* < 0.05); opposite trends were seen with GABA and 5-HT. A composite neurotransmitter score showed good prognostic accuracy, with an AUC of 0.894 (95% CI: 0.796–0.992) for mRS > 2 and 0.846 (95% CI: 0.782–0.910) for GOS < 4.

**Conclusion:**

Following microsurgical hematoma evacuation in elderly SICH patients, postoperative neurotransmitter profiles were associated with long-term functional outcomes. Persistently high Glu/NE and low GABA/5-HT levels predicted poor recovery, especially in those with large or lobar hematomas.

## 1 Introduction

Spontaneous intracerebral hemorrhage (SICH) is an acute cerebrovascular event characterized by high mortality and disability rates, accounting for 15–20% of all strokes (1). In elderly populations, factors such as increased vascular fragility, hypertension, and cerebral amyloid angiopathy substantially elevate the risk of SICH, and outcomes in this group are poorer than in younger patients (2). Despite advances in neurosurgical techniques and perioperative management in recent years, clinical treatment of SICH remains highly challenging. In particular, effective hematoma evacuation, mitigation of secondary brain injury, and promotion of neurological recovery remain critical issues in need of resolution.

Current management strategies for SICH include conservative treatment and surgical intervention. Conservative measures focus on blood pressure control, reduction of intracranial pressure, and maintenance of vital signs; however, their efficacy is limited in patients with moderate-to-large hemorrhages or impending herniation (3). Surgical options encompass conventional craniotomy for hematoma removal, minimally invasive puncture and drainage, and microsurgical techniques. Among these, microsurgery, by virtue of its precision and minimally invasive approach, allows for maximal preservation of normal brain tissue while achieving thorough hematoma evacuation, and has become an important treatment modality for SICH recently (4). Patients with cerebellar intracerebral hemorrhage (ICH) were excluded from this study because clot evacuation is rarely performed during decompressive surgery in this population. Although cerebellar and cerebral ICH share many underlying pathophysiological mechanisms—including neurotransmitter alterations contributing to secondary injury—the current evidence regarding neurotransmitter dynamics in cerebellar ICH is limited, and thus focusing on cerebral ICH enhances the consistency of intervention and outcome evaluation. Nevertheless, in elderly patients who often present with multiple comorbidities and reduced physiological reserve, the selection of surgical indications and the prospects for postoperative neurological recovery remain controversial.

Neurotransmitters are key mediators of inter-neuronal communication, and their dynamic balance is essential for normal brain function. It has been shown that, following SICH, hematoma-induced compression and ischemia-hypoxia provoke the excessive release of excitatory neurotransmitters, leading to excitotoxic neuronal injury (5). Concurrent reductions in inhibitory neurotransmitters further exacerbate neurological dysfunction (6). Accordingly, monitoring serum neurotransmitter fluctuations may offer novel biomarkers for assessing the extent of brain injury and predicting prognosis.

To date, few studies have examined the impact of microsurgical intervention on neurotransmitter dynamics in SICH patients, particularly within the elderly cohort. It remains to be determined whether surgical evacuation can effectively suppress the release of excitatory neurotransmitters, restore inhibitory neurotransmitter balance, and thereby improve neurological outcomes. Compared with conventional craniotomy, microsurgery offers the advantages of smaller incisions, clearer operative fields, and more complete hematoma removal, making it especially suitable for deep-seated hemorrhages (e.g., basal ganglia and thalamus) or those located in eloquent brain regions (7). Several clinical investigations have demonstrated that microsurgical techniques can significantly reduce mortality and enhance postoperative neurological scores in SICH patients (8). However, due to diminished cerebral compensatory capacity, elderly patients often exhibit slower postoperative recovery and greater variability in long-term outcomes. Against this backdrop, the present study aims to assess the impact of microsurgical hematoma evacuation on short-term serum neurotransmitter levels in elderly SICH patients and to explore their association with patient prognosis.

## 2 Materials and methods

### 2.1 Study participants

This single-center, prospective, and single-arm cohort study enrolled 106 elderly patients with SICH admitted between January 2023 and December 2024, all of whom underwent microsurgical hematoma evacuation. Patients were included if they: (1) aged ≥60 years; (2) diagnosed with SICH by cranial computed tomography (CT); (3) had surgical treatment performed within 24 h of admission; (4) had hematoma volume ≥10 mL, with planned microsurgical evacuation; (5) were willing to complete 180 days of postoperative follow-up, with an expected survival >3 months; and (6) provided informed consent signed by patient or legal representative, with agreement to cooperate with follow-up. Excluded individuals: (1) had secondary intracerebral hemorrhage (e.g., ruptured aneurysm, cerebral vascular malformation, tumor-related hemorrhage, or hemorrhagic transformation of cerebral infarction); (2) had severe cardiac, hepatic, renal insufficiency, or other terminal illness; (3) had active malignancy or history of chemotherapy/radiotherapy within 6 months; (4) developed major intracranial structural malformation affecting surgical approach or prognostic assessment; (5) died intraoperatively or early postoperatively (within 7 days); (6) had preexisting neurological disorders (e.g., Parkinson’s disease, Alzheimer’s disease, epilepsy, or severe sequelae of traumatic brain injury); (7) had moderate-to-severe psychiatric disorders (e.g., major depressive disorder, anxiety disorders, bipolar disorder, or schizophrenia); and (8) used drugs known to affect neurotransmitter levels within long-term; (9) had cerebellar ICH. This study was approved by the Institutional Ethics Committee and conducted in accordance with the *Declaration of Helsinki*.

### 2.2 Surgical procedure

All patients underwent microsurgical evacuation of the hematoma within 24 h of admission. Cranial CT and magnetic resonance imaging (MRI) were employed by an experienced neurosurgical team to enable precise assessment of hematoma volume, determination of its location and relationship to eloquent brain structures, comprehensive evaluation of neurological status and surgical risk, and formulation of an individualized surgical plan. Under general anesthesia, patients were positioned supine or lateral with the head fixed in a three-pin head clamp to ensure adequate exposure of the surgical field without undue pressure. An appropriate depth of anesthesia was maintained under the vigilant monitoring of the anesthesiologist to ensure the stability of the patient’s vital signs. After standard sterile preparation of the scalp and neck, a sterile drape was applied. According to the *Guidelines for the Management of Spontaneous Intracerebral Hemorrhage* from American Heart Association/American Stroke Association (9, 10) and relevant neurosurgical protocols, image-guided localization determined the skin incision and bone flap design. A high-speed craniotome was used to create the craniotomy, balancing lesion exposure against maximal preservation of normal skull integrity.

Under an operating microscope (M530 OHX, Leica, Wetzlar, Germany), the dura mater was incised to expose the cortical surface, taking care to avoid injury to meningeal vessels. Using microsurgical techniques, the hematoma cavity was gently dissected, and hematoma material was removed with suction and microbayonet forceps to minimize mechanical injury and intraoperative bleeding. Intraoperative neurophysiological monitoring, including somatosensory evoked potentials and motor evoked potentials, was used to track neural function in real time. After hematoma evacuation, meticulous hemostasis was achieved and the cavity was irrigated repeatedly with sterile normal saline. A drainage catheter was placed in the hematoma cavity or subdural space as indicated to prevent fluid accumulation or rebleeding. The dura mater was closed, the bone flap replaced and secured with titanium plates and screws, and the scalp sutured.

Postoperatively, patients were transferred immediately to the neurological intensive care unit (NICU) for strict monitoring of vital signs and neurological status, including respiratory support, hemodynamic management, cerebral edema control, and infection prophylaxis. Sedation, anticonvulsant, and neuroprotective therapies were administered as clinically indicated. Intraoperative anesthesia, surgical procedures, and postoperative care were administered by the same medical team.

### 2.3 Outcome measurement

#### 2.3.1 Surgical parameters

These surgical parameters were recorded: operative time; length of hospital stay; time to postoperative consciousness recovery; intraoperative blood loss; and hematoma evacuation rate assessed by CT within 48 h postoperatively.

#### 2.3.2 Serum neurotransmitter assessment

Venous blood samples (5 mL) were collected in the fasting state on the morning before surgery and on postoperative day 7. Serum levels of the following neurotransmitters were measured using enzyme-linked immunosorbent assay (ELISA): glutamate (Glu), γ-aminobutyric acid (GABA), norepinephrine (NE), and 5-hydroxytryptamine (5-HT). All assays were performed in a single laboratory according to the manufacturer’s instructions.

#### 2.3.3 Follow-up and long-term prognosis

In addition to the 180-day follow-up evaluation, modified Rankin Scale (mRS) scores were also recorded at the time of hospital discharge for all patients. This allowed for evaluation of short-term functional changes and comparison with long-term recovery. Patients were followed for 180 days via hospitalization records, outpatient visits, and telephone interviews. Complications (e.g., rebleeding, infection, or hydrocephalus) and mortality were recorded. At postoperative day 180, neurological function was evaluated using the modified Rankin Scale (mRS) and the Glasgow Outcome Scale (GOS), in which higher mRS scores indicate greater disability, whereas higher GOS scores denote better functional recovery. Subgroup analyses were conducted based on hematoma volume (<30 mL vs. ≥30 mL), hemorrhage location (basal ganglia, thalamus, or lobar regions), and preoperative Glasgow Coma Scale (GCS) score (≤8 vs. >8) to compare neurotransmitter changes and long-term outcomes among groups.

### 2.4 Statistical analysis

Data were analyzed using SPSS version 26.0 (IBM Corp., Armonk, NY, USA) and R (R Foundation for Statistical Computing, Vienna, Austria). Continuous variables were tested for normality. Normally distributed data are presented as $\bar{x}$ ± *s*, with paired *t*-tests for within-group comparisons and independent *t*-tests or one-way analysis of variance (ANOVA) for subgroup comparisons. Non-normally distributed data are expressed as median [M (P25, P75)] and compared using the Wilcoxon signed-rank test for intra-group comparison, or the Mann–Whitney U test/Kruskal–Wallis test for inter-group comparison. Categorical variables are presented as frequencies and percentages, with between-group comparisons by *χ*^2^ test or Fisher’s exact test. Pearson correlation analysis was used to assess relationships between postoperative neurotransmitter changes and long-term functional scores. A two-sided *p*-value <0.05 was considered statistically significant.

## 3 Results

### 3.1 Patient baseline characteristics

A total of 106 elderly patients with SICH were enrolled, including 72 men (67.9%) and 34 women (32.1%). The mean age was 70.55 ± 6.01 years and the mean body mass index (BMI) was 22.93 ± 4.10 kg/m^2^. The average hematoma volume was 28.84 ± 9.42 mL, and the mean preoperative GCS score was 11.19 ± 3.48. Comorbidities included hypertension (*n* = 74), diabetes (*n* = 29), atrial fibrillation (*n* = 26), coronary artery disease (*n* = 24), and a history of prior stroke (*n* = 17).

### 3.2 Surgical parameter data

The mean operative time was 119.36 ± 23.49 min. The average length of hospital stay was 14.94 ± 5.26 days. The time to postoperative consciousness recovery averaged 17.33 ± 6.30 h. Mean intraoperative blood loss was 31.71 ± 15.72 mL. The hematoma evacuation rate on 48-h postoperative CT was 86.45% ± 7.00%.

### 3.3 Changes in serum neurotransmitters

**Figure 1:**
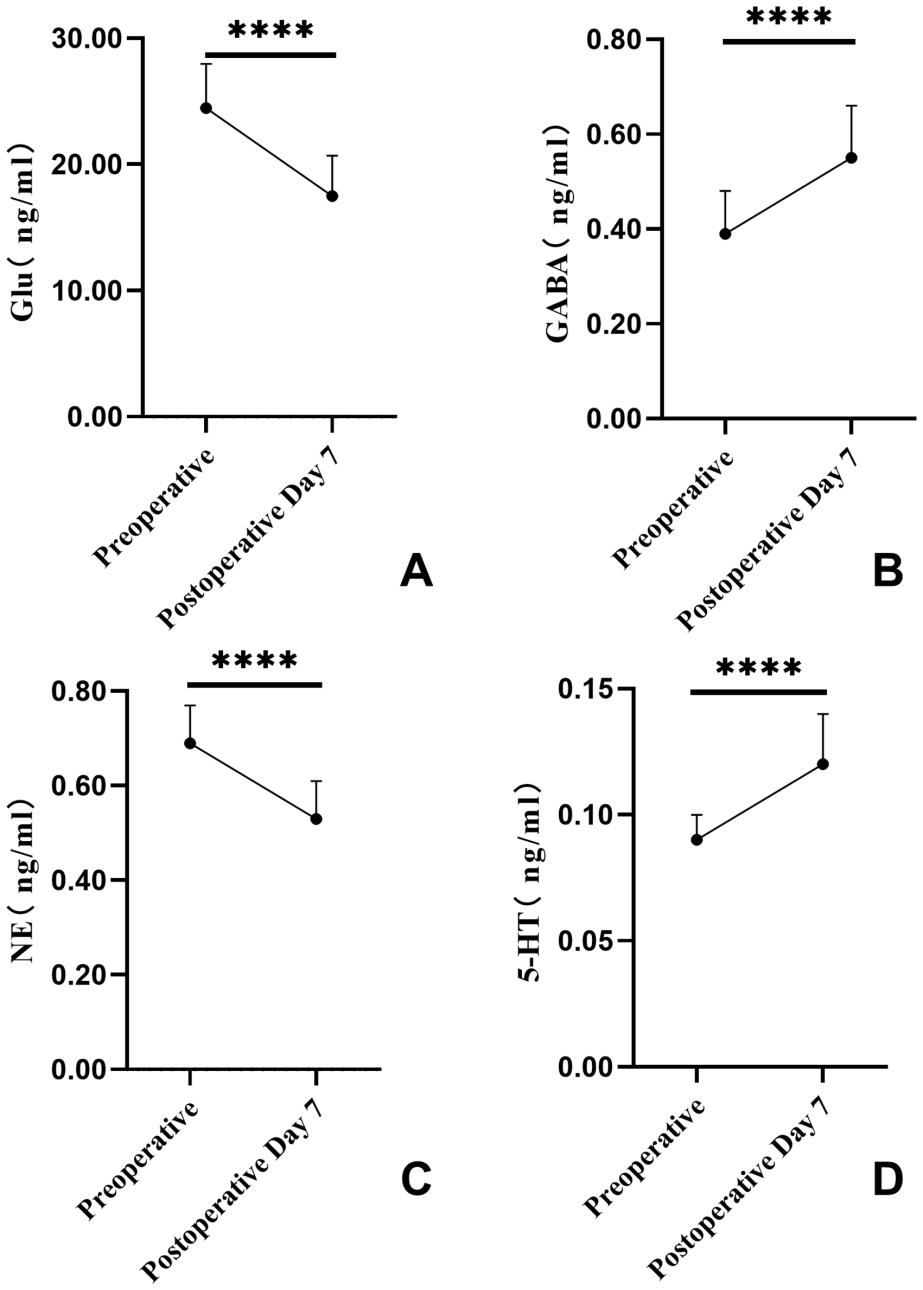
Pre- and postoperative trends in serum neurotransmitter levels. **(A–D)** Comparisons of preoperative versus postoperative day 7 levels of Glu, GABA, NE, and 5-HT; **** denotes *p* < 0.0001.

From preoperative to postoperative day 7, Glu and NE levels decreased significantly, while GABA and 5-HT levels increased significantly (all *p* < 0.05) (Table 1; Figure 1).

**Table 1:** Pre- and postoperative serum neurotransmitter level changes ( $\bar{x}\pm s$ , ng/mL).

Parameters	Preoperative	Postoperative day 7	*t*	*p*
Glu	24.46 ± 3.51	17.48 ± 3.20	61.372	0.000
GABA	0.39 ± 0.09	0.55 ± 0.11	−42.018	0.000
NE	0.69 ± 0.08	0.53 ± 0.08	50.447	0.000
5-HT	0.09 ± 0.01	0.12 ± 0.02	−33.624	0.000

### 3.4 Prognosis outcome

**Figure 2:**
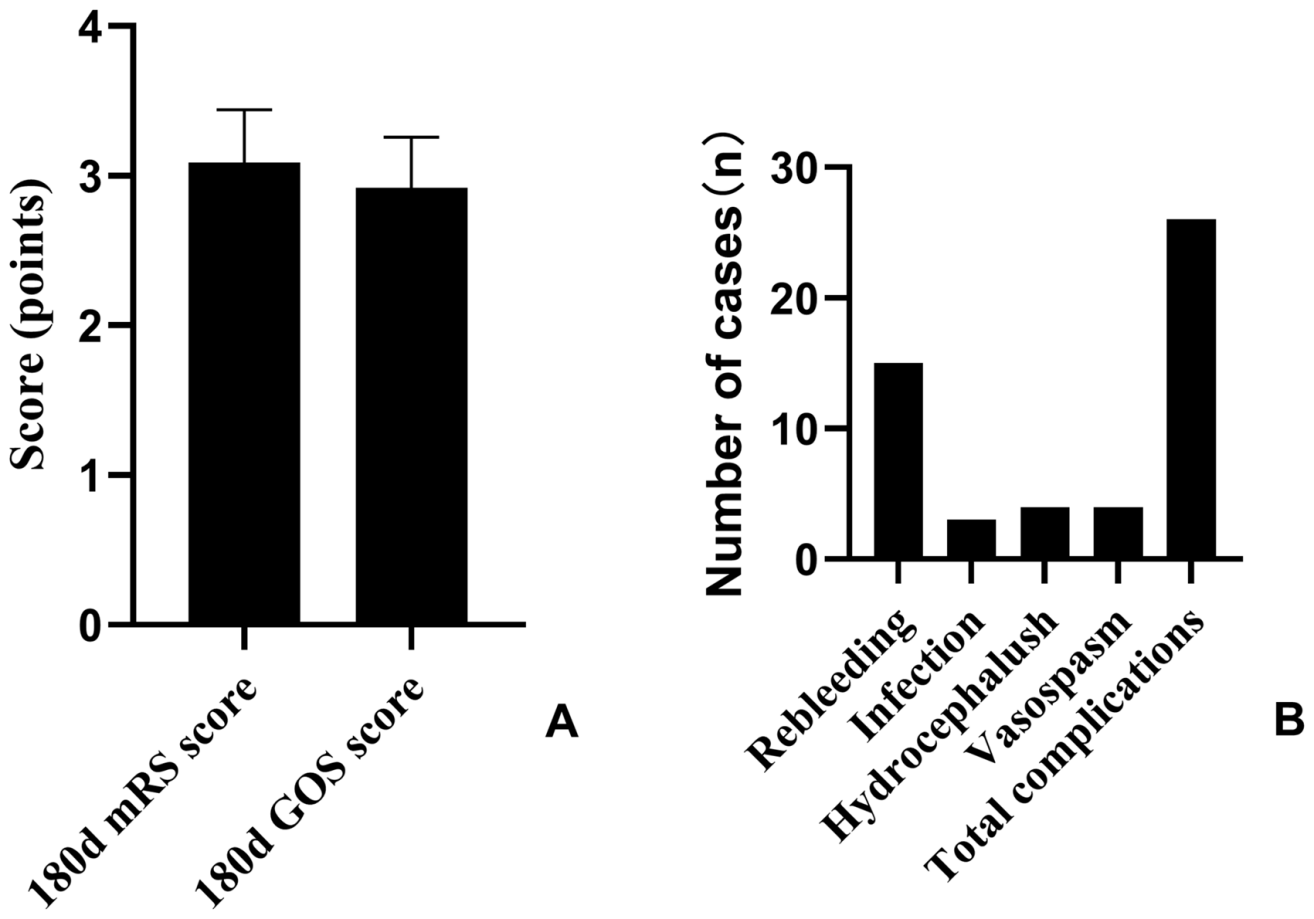
Neurological functional outcomes and complications at 180 days postoperatively. **(A)** Distribution of mRS and GOS scores at 180 days after surgery. **(B)** Incidence of postoperative complications.

At postoperative day 180, there were no deaths. The mean mRS score was 3.09 ± 0.35, indicating that most patients had regained a good degree of independence. The mean GOS score was 2.92 ± 0.34, reflecting overall favorable neurological recovery. Postoperative complications occurred in 26 patients (24.5%): 15 rebleeds, 3 infections, 4 cases of hydrocephalus, and 4 instances of cerebral vasospasm (Figure 2).

### 3.5 Comparison of postoperative day 7 serum neurotransmitter levels and 180-day long-term neurological function indicators among subgroups

Subgroup analyses were performed based on hematoma volume, hemorrhage location, and preoperative GCS score. When stratified by hematoma volume, 37 patients had volumes ≥30 mL and 69 had volumes <30 mL. The hematoma (≥30 mL) group demonstrated significantly higher Glu, NE levels, and mRS scores than the hematoma (<30 mL) group, whereas GABA and 5-HT levels and GOS scores were significantly lower (*p* < 0.05). By hemorrhage location, 53 patients (49.6%) had basal ganglia hemorrhages, 32 (30.2%) had thalamic hemorrhages, and 21 (20.2%) had lobar hemorrhages. Patients with lobar hemorrhages exhibited significantly higher Glu and NE levels and lower GABA and 5-HT levels compared with both basal ganglia and thalamic groups (*p* < 0.05). Additionally, the lobar group had a significantly lower GOS score than the thalamic group, although mRS scores did not differ significantly among the three locations (*p* > 0.05). According to preoperative GCS score, 53 patients (49.6%) were classified as high score (13–15), 32 (30.2%) as middle score (9–12), and 21 (20.2%) as low score (3–8). The middle-score group (GCS 9–12) had the highest Glu levels and lowest GABA levels, while the low-score group (GCS 3–8) showed significantly lower 5-HT levels (*p* < 0.05). Moreover, the middle-score group had the worst mRS and lowest GOS scores, indicating the poorest neurological recovery among the three groups (Table 2).

**Table 2:** Comparison of day 7 postoperative serum neurotransmitter levels and 180-day outcomes across subgroups.

Subgroups		Glu (ng/mL)	GABA (ng/mL)	NE (ng/mL)	5-HT (ng/mL)	mRS (scores)	GOS (scores)
Hematoma volume	≥30 mL (*n* = 37)	20.70 ± 2.29	0.43 ± 0.04	0.62 ± 0.06	0.11 ± 0.02	3.24 ± 0.44	2.76 ± 0.44
	<30 mL (*n* = 69)	15.75 ± 2.09	0.62 ± 0.09	0.48 ± 0.04	0.12 ± 0.01	3.01 ± 0.27	3.00 ± 0.24
	*t*	11.237	−12.374	16.183	−2.034	3.333	−3.703
	*p*	0.000	0.000	0.000	0.045	0.000	0.000
Hematoma location	Basal ganglia, (*n* = 53)	17.13 ± 3.44	0.57 ± 0.12	0.51 ± 0.08	0.12 ± 0.02	3.08 ± 0.39	2.92 ± 0.38
	Thalamus (*n* = 32)	16.48 ± 2.04	0.58 ± 0.09	0.51 ± 0.05	0.12 ± 0.01	3.06 ± 0.25	3.00 ± 0.01
	Lobar (*n* = 21)	19.89 ± 2.95	0.47 ± 0.08	0.60 ± 0.08	0.10 ± 0.01	3.19 ± 0.40	2.76 ± 0.44
	*F*	8.983	8.777	12.293	19.747	0.986	3.259
	*p*	0.000	0.000	0.000	0.000	0.376	0.042
Preoperative GCS scores	High (13–15, *n* = 53)	16.38 ± 2.58	0.59 ± 0.11	0.52 ± 0.07	0.12 ± 0.01	3.03 ± 0.31	3.03 ± 0.18
	Medium (9–12, *n* = 32)	19.97 ± 2.93	0.47 ± 0.07	0.55 ± 0.10	0.12 ± 0.01	3.24 ± 0.44	2.69 ± 0.47
	Low (3–8, *n* = 21)	17.28 ± 3.58	0.57 ± 0.12	0.54 ± 0.10	0.09 ± 0.01	3.07 ± 0.27	2.86 ± 0.36
	*F*	16.120	11.976	1.471	79.398	3.727	12.424
	*p*	0.000	0.000	0.234	0.000	0.027	0.000

### 3.6 Correlation between postoperative day 7 neurotransmitter levels and 180-day outcomes

**Figure 3:**
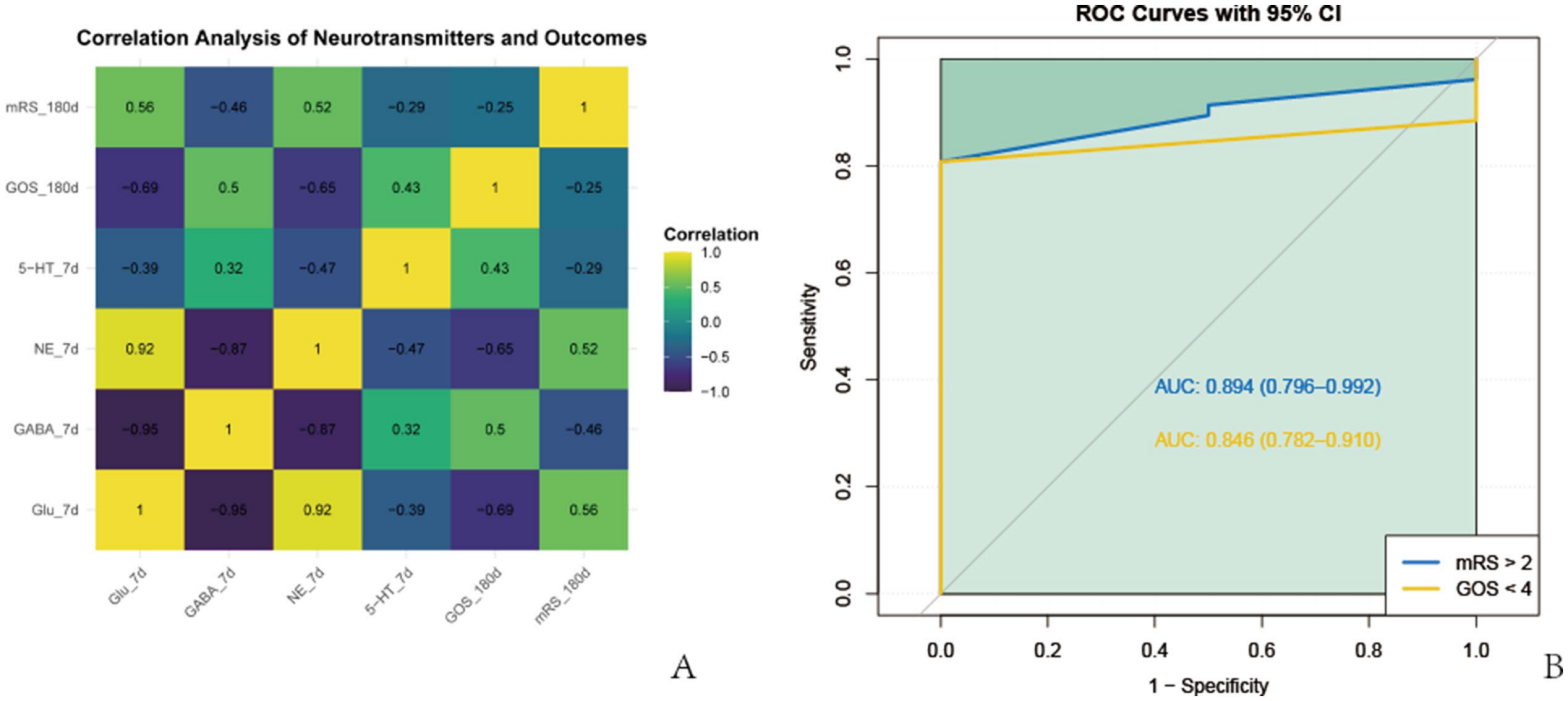
Correlation and prognostic value of serum neurotransmitters. **(A)** Pearson correlation analysis between serum neurotransmitter levels on postoperative day 7 and neurological functional outcomes at 180 days (e.g., mRS and GOS scores). **(B)** Receiver operating characteristic (ROC) curves assessing the predictive performance of neurotransmitter-based composite scores for poor neurological outcomes (defined as mRS > 2 or GOS < 4).

Pearson correlation analysis was performed to assess the relationships between postoperative neurotransmitter level changes and long-term neurological function scores. On postoperative day 7, Glu and NE levels exhibited significant positive correlations with mRS scores (*p* < 0.05), whereas GABA and 5-HT levels demonstrated significant negative correlations with mRS scores (*p* < 0.05). Conversely, Glu and NE levels correlated negatively with GOS scores (*p* < 0.05), while GABA and 5-HT levels correlated positively with GOS scores (*p* < 0.05) (Figure 3A). To further evaluate the prognostic value of these neurotransmitter alterations, a composite neurotransmitter score was calculated and subjected to ROC curve analysis. The score showed good predictive performance for poor neurological outcomes at 180 days. For mRS > 2, the area under the curve (AUC) was 0.894 (95% CI: 0.796–0.992); for GOS < 4, the AUC was 0.846 (95% CI: 0.782–0.910) (Figure 3B).

## 4 Discussion

The pathophysiological process of SICH is divided into primary and secondary injury phases. Primary injury is induced predominantly by the mass effect of the hematoma, resulting in compression of adjacent brain tissue, increased intracranial pressure, and reduced cerebral perfusion (11). Secondary injury involves complex molecular mechanisms, including inflammatory responses, oxidative stress, blood–brain barrier disruption, and excitotoxicity mediated by excitatory amino acids such as Glu (12). Together, these factors precipitate neuronal apoptosis and neurological deficits, severely compromising patient quality of life and long-term prognosis. With the aging of China’s population, the incidence of SICH in the elderly has risen, imposing a substantial burden on both society and families. Existing studies have demonstrated that direct hematoma mass effect, secondary edema, and inflammatory cascades are the principal drivers of post-hemorrhagic neurological injury (13). Alterations in neurotransmitter levels, particularly Glu and GABA, have been shown to correlate closely with the extent of tissue injury and functional recovery, suggesting their potential utility as early biological markers. Conservative management affords some benefit in blood-pressure control, intracranial pressure reduction, and vital-sign support but provides limited efficacy in hematoma evacuation. In recent years, microsurgical hematoma evacuation has been increasingly adopted for SICH treatment owing to its minimal invasiveness, precise technique, and capacity for thorough clot removal with reduced collateral tissue damage. This approach has been particularly advocated for patients presenting with moderate-to-large hematomas or impending herniation. Nonetheless, the effects of microsurgery on short-term serum neurotransmitter dynamics and long-term neurological recovery in elderly SICH patients remain inadequately characterized.

In the present study of 106 elderly SICH patients undergoing microsurgical evacuation, significant postoperative reductions in Glu and NE levels and concomitant elevations in GABA and 5-HT levels were observed at postoperative day 7. These findings suggest that, beyond its anatomic decompressive effects, microsurgical clot removal may facilitate restoration of neurotransmitter homeostasis at the molecular level, thereby improving neurological outcomes.

Glu and NE represent the principal excitatory neurotransmitters in the brain and are known to rise markedly following SICH, contributing to excitotoxicity, cerebral edema, blood–brain barrier disruption, and activation of inflammatory mediators. Elevated plasma Glu levels in SICH patients has been reported to be closely associated with early postoperative edema formation and poor prognosis (14), while other investigators have demonstrated that NE-mediated sympathetic overactivation exacerbates secondary brain injury (15). The significant reductions in Glu and NE levels observed in this study at postoperative day 7 implied that precise hematoma evacuation and mechanical decompression afforded by microsurgery may attenuate the persistent release of excitatory transmitters and inhibit secondary neuronal injury. These results align with those scholars who found that stereotactic clot evacuation reduced Glu levels and improved acute consciousness states (16), further underscoring the mechanistic importance of early surgical intervention in controlling excitotoxicity. By contrast, GABA and 5-HT are prototypical inhibitory neurotransmitters whose postoperative elevations may reflect activation of endogenous neuroprotective mechanisms that buffer excitotoxic damage and promote neural plasticity and reconnection. Activation of the 5-HT signaling pathway after intracerebral hemorrhage correlated with accelerated functional recovery (17), and GABA upregulation has been linked to reduced inflammatory mediators and preservation of eloquent cortex.

Subgroup analysis elucidated key clinical factors influencing surgical efficacy. Patients with hematoma volumes ≥30 mL exhibited higher Glu and NE levels and poorer outcomes, indicating that hematoma load directly impacts postoperative neurotransmitter metabolism and functional recovery. Larger bleeds produce more extensive tissue compression and destruction, triggering more severe Glu excitotoxicity and NE-related stress responses. This imbalance exacerbates secondary injury, disrupts neural network remodeling, and impairs long-term recovery. Conversely, smaller hematomas were associated with more moderate neurotransmitter shifts and better functional prognosis, suggesting hematoma volume as a critical predictor of postoperative metabolic re-equilibration and outcome. By hemorrhage location, the lobar group manifested the highest Glu and NE levels, lowest GABA and 5-HT levels, and a significantly lower GOS score compared with the thalamic group. The cerebral lobes, particularly frontal and temporal regions, subserve motor, language, and higher cognitive functions. Lobar hemorrhages, though less frequent than basal ganglia or thalamic bleeds, produce more direct cortical disruption and consequent deficits. The elevated excitatory (Glu and NE) and reduced inhibitory transmitter (GABA and 5-HT) profile indicated excessive local neuronal activation and dysregulation, underscoring the need for early intensive intervention and rehabilitation. Additionally, patients with moderate preoperative GCS scores (9–12) fared worst across multiple parameters, suggesting that moderate impairment may reflect a precarious balance between primary and secondary injury. This finding is not entirely consistent with the linear relationship between lower GCS scores and poorer prognosis reported in previous research (18). One possible explanation is that patients with moderate impairment (GCS 9–12) may not exhibit as severe a clinical presentation as those in deep coma (GCS 3–8), and therefore may not receive as prompt initiation of therapeutic interventions, while also lacking the relative stability observed in patients with mild impairment (GCS 13–15). Such patients may exist in a state of pathological compensation, during which cerebral edema and metabolic dysregulation progressively worsen, while excitatory neurotransmitter release remains elevated, and inhibitory neurotransmitter regulation is insufficient. In this “pre-decompensation” phase, disease progression may be misjudged or treatment delayed, predisposing these patients to postoperative extension of secondary injury and resulting in the poorest long-term neurological recovery.

mRS and GOS are standard tools for prognostic assessment, reflecting functional independence and global neurological recovery, respectively. At postoperative day 180, overall outcomes were favorable. Strong correlations were identified between postoperative day 7 neurotransmitter levels and long-term mRS and GOS scores, which suggested that microsurgical hematoma evacuation may facilitate the restoration of neurological function in elderly SICH patients, and that acute-phase alterations in neurotransmitter levels may serve as reliable predictors of long-term neurological recovery. Specifically, higher Glu and NE levels predicted higher mRS and lower GOS scores, whereas elevated GABA and 5-HT levels were associated with better outcomes. These correlations highlight the enduring impact of acute-phase neurochemical milieu on subsequent neural network remodeling and functional restoration. Excessive postoperative excitatory neurotransmission promotes calcium influx, mitochondrial dysfunction, and free-radical generation, exacerbating neuron loss and sustaining secondary injury (19, 20). Failure of timely reduction in their concentrations may portend ongoing tissue damage and compromised recovery. Previous animal study has similarly linked persistent Glu elevation with perihematomal metabolic disturbances and delayed apoptosis (21).

Conversely, GABA and 5-HT, as central inhibitory modulators, exert anti-excitotoxic, anti-inflammatory, and neuroplasticity-promoting effects. Patients exhibiting postoperative day 7 increases in GABA and 5-HT demonstrated significantly better 180-day functional recovery, indicating that activation of inhibitory pathways may create a permissive environment for tissue repair and network reconstitution. This observation concurs with reports of 5-HT-mediated axonal regeneration and functional improvement (22), and it supports exploration of GABAergic enhancement therapies in brain-injury rehabilitation. Thus, postoperative neurotransmitter profiling may serve not only as a biomarker of injury evolution but also as a potential target for tailored long-term interventions, such as early Glu/NE antagonists and GABA/5-HT augmenting agents, to optimize outcomes in elderly SICH patients. Although serum measurements cannot fully reflect central neurotransmitter dynamics due to the blood–brain barrier and peripheral metabolic influences, prior studies have shown meaningful correlations between serum and cerebrospinal fluid (CSF) levels of key neurotransmitters such as Glu and GABA in acute brain injury (23). Therefore, serum profiles may still provide clinically relevant insights into central neurochemical status and functional recovery.

Additionally, ROC analysis demonstrated that the combined neurotransmitter score robustly predicted poor neurological outcomes, with AUCs of 0.894 for mRS > 2 and 0.846 for GOS < 4, supporting its value as a prognostic biomarker.

## 5 Research significance and limitations

This study has several limitations. First, as a single-center and prospective observational investigation with a limited sample size, the findings may not fully capture the heterogeneity of the broader patient population. Second, although serum neurotransmitter levels provide valuable insight into cerebral metabolic status, they cannot be assumed to directly mirror central nervous system neurotransmitter dynamics. Future studies should incorporate cerebrospinal fluid assays or metabolic neuroimaging modalities for validation. Third, postoperative interventions, such as rehabilitative therapy and pharmacological support, were not uniformly controlled, which may have introduced confounding effects on long-term functional outcomes. Fourth, although mRS scores at hospital discharge were systematically recorded, they were not included in detailed analysis. This decision was made to maintain consistency with the study’s primary objective—evaluating long-term neurological function at 180 days. Discharge mRS scores may be influenced by transient perioperative factors, variable hospital stay durations, and early complications, potentially limiting their ability to reflect true recovery status. By focusing on the 180-day mRS, the study aimed to provide a more stable and clinically meaningful assessment of functional outcome. Nevertheless, future studies incorporating serial mRS assessments at multiple early and late time points may offer a more comprehensive understanding of recovery trajectories following microsurgical hematoma evacuation.

## 6 Conclusion

Postoperative shifts in serum neurotransmitter levels, particularly elevated Glu and NE alongside reduced GABA and 5-HT, were consistently associated with poorer long-term neurological outcomes in this cohort. These associations remained evident across correlation and ROC analyses, suggesting that neurotransmitter profiles may reflect the underlying neurophysiological status after hematoma evacuation. While the present findings are limited by the absence of a non-surgical control group, they offer a basis for further investigation into the utility of serum neurotransmitters as adjunctive indicators in postoperative assessment and prognostic stratification.

## Data Availability

The original contributions presented in the study are included in the article/supplementary material, further inquiries can be directed to the corresponding author.

## References

[1] Jain A Malhotra A Payabvash S. Imaging of spontaneous intracerebral hemorrhage. Neuroimaging Clin N Am. (2021) 31:193–203. doi: 10.1016/j.nic.2021.02.003, PMID: 33902874 PMC8820948

[2] Sheth KN. Spontaneous intracerebral hemorrhage. N Engl J Med. (2022) 387:1589–96. doi: 10.1056/NEJMra2201449, PMID: 36300975

[3] Al-Kawaz MN Hanley DF Ziai W. Advances in therapeutic approaches for spontaneous intracerebral hemorrhage. Neurotherapeutics. (2020) 17:1757–67. doi: 10.1007/s13311-020-00902-w, PMID: 32720246 PMC7851203

[4] Ma H Peng W Xu S Liang X Zhao R Lv M. Advancements of endoscopic surgery for spontaneous intracerebral hemorrhage. World Neurosurg. (2025) 193:160–70. doi: 10.1016/j.wneu.2024.10.107, PMID: 39491620

[5] Snider S Albano L Gagliardi F Comai S Roncelli F De Domenico P. Substantially elevated serum glutamate and CSF GOT-1 levels associated with cerebral ischemia and poor neurological outcomes in subarachnoid hemorrhage patients. Sci Rep. (2023) 13:5246. doi: 10.1038/s41598-023-32302-3, PMID: 37002262 PMC10066256

[6] Liu C He P Guo Y Tian Q Wang J Wang G. Taurine attenuates neuronal ferroptosis by regulating GABA(B)/AKT/GSK3β/β-catenin pathway after subarachnoid hemorrhage. Free Radic Biol Med. (2022) 193:795–807. doi: 10.1016/j.freeradbiomed.2022.11.003, PMID: 36402441

[7] Jha VC Alam MS Sinha VS. Comparative outcome of endovascular embolization with microsurgery in managing acute spontaneous cerebral hemorrhage in pediatric patients, an institutional experience. Childs Nerv Syst. (2023) 39:963–74. doi: 10.1007/s00381-022-05785-0, PMID: 36571597

[8] Schartz D Mattingly TK Rahmani R Ellens N Akkipeddi SMK Bhalla T. Noncurative microsurgery for cerebral aneurysms: a systematic review and meta-analysis of wrapping, residual, and recurrence rates. J Neurosurg. (2022) 137:129–39. doi: 10.3171/2021.9.Jns211698, PMID: 34798602

[9] Hemphill JC 3rd Greenberg SM Anderson CS Becker K Bendok BR Cushman M. Guidelines for the Management of Spontaneous Intracerebral Hemorrhage: a guideline for healthcare professionals from the American Heart Association/American Stroke Association. Stroke. (2015) 46:2032–60. doi: 10.1161/str.0000000000000069 26022637

[10] Greenberg SM Ziai WC Cordonnier C Dowlatshahi D Francis B Goldstein JN. 2022 guideline for the management of patients with spontaneous intracerebral hemorrhage: a guideline from the American Heart Association/American Stroke Association. Stroke. (2022) 53:e282–361. doi: 10.1161/str.0000000000000407, PMID: 35579034

[11] Rossi J Hermier M Eker OF Berthezene Y Bani-Sadr A. Etiologies of spontaneous acute intracerebral hemorrhage: a pictorial review. Clin Imaging. (2023) 95:10–23. doi: 10.1016/j.clinimag.2022.12.007 36577316

[12] Liu M Wang Z Meng X Zhou Y Hou X Li L. Predictive nomogram for unfavorable outcome of spontaneous intracerebral hemorrhage. World Neurosurg. (2022) 164:e1111–22. doi: 10.1016/j.wneu.2022.05.111, PMID: 35654327

[13] van Etten ES Kaushik K Jolink WMT Koemans EA Ekker MS Rasing I. Trigger factors for spontaneous intracerebral hemorrhage: a case-crossover study. Stroke. (2022) 53:1692–9. doi: 10.1161/strokeaha.121.036233, PMID: 34911344

[14] Kawakita F Kanamaru H Asada R Suzuki Y Nampei M Nakajima H. Roles of glutamate in brain injuries after subarachnoid hemorrhage. Histol Histopathol. (2022) 37:1041–51. doi: 10.14670/hh-18-509 36065974

[15] Lakhal K Hivert A Alexandre PL Fresco M Robert-Edan V Rodie-Talbere PA. Intravenous Milrinone for cerebral vasospasm in subarachnoid hemorrhage: the MILRISPASM controlled before-after study. Neurocrit Care. (2021) 35:669–79. doi: 10.1007/s12028-021-01331-z, PMID: 34478028

[16] Wu G Li S Wang L Mao Y. The perihematomal glutamate level is associated with the outcome of patients with basal ganglia hematomas treated by minimally invasive procedures. Neurol Res. (2013) 35:829–36. doi: 10.1179/1743132813y.0000000220, PMID: 23676149

[17] Keins S Abramson JR Mallick A Castello JP Rodriguez-Torres A Popescu D. Association of depression onset and treatment with blood pressure control after intracerebral hemorrhage. Stroke. (2023) 54:105–12. doi: 10.1161/strokeaha.122.040331, PMID: 36444719 PMC11755381

[18] Xu X Zhang H Zhang J Luo M Wang Q Zhao Y. Minimally invasive surgeries for spontaneous hypertensive intracerebral hemorrhage (MISICH): a multicenter randomized controlled trial. BMC Med. (2024) 22:244. doi: 10.1186/s12916-024-03468-y, PMID: 38867192 PMC11170771

[19] Dong YJ Jiang NH Zhan LH Teng X Fang X Lin MQ. Soporific effect of modified Suanzaoren decoction on mice models of insomnia by regulating orexin-a and HPA axis homeostasis. Biomed Pharmacother. (2021) 143:112141. doi: 10.1016/j.biopha.2021.112141, PMID: 34509822

[20] Sears SM Hewett SJ. Influence of glutamate and GABA transport on brain excitatory/inhibitory balance. Exp Biol Med (Maywood). (2021) 246:1069–83. doi: 10.1177/1535370221989263, PMID: 33554649 PMC8113735

[21] Czapski GA Strosznajder JB. Glutamate and GABA in microglia-neuron cross-talk in Alzheimer's disease. Int J Mol Sci. (2021) 22:11677. doi: 10.3390/ijms222111677, PMID: 34769106 PMC8584169

[22] Borroto-Escuela DO Ambrogini P Chruścicka B Lindskog M Crespo-Ramirez M Hernández-Mondragón JC. The role of central serotonin neurons and 5-HT Heteroreceptor complexes in the pathophysiology of depression: a historical perspective and future prospects. Int J Mol Sci. (2021) 22:1927. doi: 10.3390/ijms22041927, PMID: 33672070 PMC7919680

[23] Elander A Gustafsson M. Inhaler technique and self-reported adherence to medications among hospitalised people with asthma and COPD. Drugs Real World Outcomes. (2020) 7:317–23. doi: 10.1007/s40801-020-00210-x, PMID: 33052539 PMC7581666

